# Chronic Tic Disorders in Youth: Clinical Phenotypes and Response to Pharmacological Treatment with Aripiprazole

**DOI:** 10.3390/children11121459

**Published:** 2024-11-29

**Authors:** Francesca Falcone, Stefano Berloffa, Gianluca Sesso, Antonio Narzisi, Elena Valente, Valentina Viglione, Gabriele Masi, Annarita Milone, Pamela Fantozzi

**Affiliations:** 1 Developmental Psychiatry and Psycopharmacology Unit, IRCCS Stella Maris Foundation, 311 viale del Tirreno, 56018 Pisa, Italy; francesca.falcone@fsm.unipi.it (F.F.); stefano.berloffa@fsm.unipi.it (S.B.); gianluca.sesso@fsm.unipi.it (G.S.); antonio.narzisi@fsm.unipi.it (A.N.); elena.valente@fsm.unipi.it (E.V.); valentina.viglione@fsm.unipi.it (V.V.); gabriele.masi@fsm.unipi.it (G.M.); pamela.fantozzi@fsm.unipi.it (P.F.); 2 IMT School for Advanced Studies, 19 Piazza San Francesco, 55100 Lucca, Italy

**Keywords:** tic disorders, attention deficit hyperactivity disorder, autism spectrum disorder, Aripiprazole

## Abstract

Background/Objectives: Tic disorders are neurodevelopmental conditions often associated with comorbidities like attention deficit hyperactivity disorder (ADHD) and autism spectrum disorder (ASD). Our aims were: (a) in a sample of youth with tic disorders to explore the clinical and psychopathological characteristics of different phenotypes based on the presence of comorbid ADHD and/or ASD and gender; (b) in a subgroup of patients treated with Aripiprazole, to evaluate symptoms variation over time and to identify potential predictors of response. Methods: A total of 95 subjects with tic disorders (age range 6 to 17.9 years, mean 11.1 ± 2.11 years, 80 males) were naturalistically recruited. Questionnaires and semi-structured interviews were administered to assess the symptomatology and investigate the presence of psychiatric comorbidities (Clinic Global Impression-Severity (CGI-S), Children’s Global Assessment Scale (C-GAS), Yale Global Tic Severity Scale (YGTSS), Premonitory Urge for Tics Scale (PUTS), Child Yale–Brown Obsessive Compulsive Scale for Children (CYBOCS), Child Behavior Checklist 6–18 (CBCL 6–18), Conners’ Parent Rating Scale-Revised—short form (CRSR-S), Reactivity Intensity Polarity Stability Questionnaire—youth version (RIPoSt-Y), and Social Communication Questionnaire—lifetime version (SCQ); Autism Diagnostic Observation Scale—second version (ADOS-2) and Autism Diagnostic Interview—revised version (ADI-R) were administered where ASD was suspected). A total of 22 subjects treated with Aripiprazole were reassessed through the use of some of the clinical measures used at baseline. Results: The presence of ADHD was associated with higher externalizing problem scores on the CBCL 6–18, while ASD was linked to higher internalizing problem scores. A positive correlation was found between the ADHD–ASD interaction and increased internalizing symptoms on CBCL 6–18 and higher ADOS-2 scores. Patients treated with Aripiprazole showed significant improvement across all scales during follow-up. ADHD was identified as a negative predictor of reduced tic severity on the YGTSS. Conclusions: Comorbid neurodevelopmental disorders, such as ADHD or ASD, result in worse emotional and behavioral functioning in patients with tic disorders. ADHD–ASD interaction may be linked to more internalizing symptoms and autistic behaviors. Aripiprazole improves overall clinical outcomes, although comorbid ADHD may hinder the reduction of tic symptoms.

## 1. Introduction

Tourette’s syndrome (TS) and persistent (chronic) motor or vocal tic disorder are neurodevelopmental disorders characterized by the presence of tics (motor and/or vocal tics), which may fluctuate in frequency, persisting for a period longer than 12 months [1]. The prevalence rate of TS ranges from 0.1% to 6%, with a higher incidence in males [2,3,4,5]. The onset of tics occurs between 4 and 6 years, reaching maximum interference between 10 and 12 years [6]. Generally, motor tics appear first, start with simple facial tics, progress in a rostro-caudal direction, and extend to other areas [7]. Vocal tics usually appear 1–2 years later, starting as simple tics and then becoming complex [8].

Between 85% and 90% of subjects with TS present with psychiatric comorbidities, most frequently attention-deficit/hyperactivity disorder (ADHD) and obsessive–compulsive disorder (OCD) [9,10]. Patients with TS present a lifetime prevalence of ADHD of 54.3%, with a higher incidence in males [11]; children with TS and ADHD showed greater externalizing and internalizing behavioral problems and poorer social adjustment [12] lower IQ [13], and a high prevalence of specific learning disorders [14,15] than children with TS without ADHD. OCD is the second most frequently occurring comorbidity with tic disorders. Subjects with tic disorders usually exhibit specific OCD symptoms, including obsessions related to aggressive or inappropriate sexual thoughts [16], along with counting, ordering, symmetry, or hoarding compulsions [17]. In patients with TS, the prevalence of autism spectrum disorder (ASD) ranges from 2.9% to 20% [18,19,20,21], and higher rates are documented in children [22]. Few studies have evaluated the impact of ASD within the clinical profile of patients with TS, while a larger number of studies analyzed the inverse relationship [23,24,25,26]. In patients with TS and comorbid ASD, the rate of additional comorbidities seems to be significantly higher (98.8% vs. 13.2%) [27]. Darrow et al. [22] showed that although ASD rates appear to be somewhat elevated over population rates in TS, at least some of the ASD-like symptoms (particularly repetitive behaviors) are more strongly related to comorbid symptoms, in particular, OCD symptoms.

A recent review [28] analyzing gender differences in movement disorders showed that tic manifestations (type, number, frequency, and complexity) do not exhibit significant differences between sexes in TS, although an increased frequency of tics during the estrogenic phase of the menstrual cycle has been reported [29]. Compared to males, female patients seem to have a less widespread distribution of motor tics in adulthood [30]. The onset of tic compulsions appears to be more typical in female patients than in males, while the onset of behavioral problems is more frequent in males [31]. Compared to women with TS, men more frequently show anger, which is associated with a higher prevalence of ADHD [31,32], whereas women frequently report a history of depression and anxiety [33]. Compared to males, girls with TS may have a later peak of symptoms, less remission with age, and worsening of symptoms due to tics, particularly in adulthood, reporting higher rates of tic-related interference in their social, recreational, and domestic activities [33].

Pharmacological treatments approved by the major drug agencies in the USA and Europe for patients with TS with moderate to severe functional impairment are Aripiprazole (age ≥ 6 years), Risperidone (age ≥ 7 years), and Pimozide (age ≥ 12 years).

Few clinical studies specifically address the treatment of the most common psychiatric comorbidities in patients with TS, such as ADHD and OCD [34].

Some studies documented a reduction of tics in children with tics plus ADHD treated with methylphenidate [35,36]. Gerasch et al. suggested that Aripiprazole may play a role in improving ADHD, OCD, or affective symptoms in these subjects [37]. A longitudinal study, which evaluated the use of Aripiprazole in a group of 28 patients with a primary diagnosis of TS and comorbid ADHD, showed that the medication could represent a potential therapeutic option among other possible monotherapies targeting both tic disorders and ADHD [38]. In their randomized controlled trial (RCT), Sallee et al. confirmed an improvement in ADHD symptoms in TS patients treated with higher doses of Aripiprazole [39].

From a pharmacological perspective, OCD related to tic disorders seems to respond only partially to selective serotonin reuptake inhibitors alone, compared to OCD alone [40,41]. In a comparative naturalistic study conducted on children with OCD and tic disorders, augmentation with Risperidone or Aripiprazole has proven effective in improving OCD symptoms and tic severity [42]. A retrospective study evaluating the effect of Aripiprazole in 37 patients with TS and impulse control disorders [43], followed for 12 weeks, demonstrated that subjects treated with Aripiprazole showed a reduction in tic severity for the entire study period.

Thus, the aims of our study were (1) to explore the clinical and psychopathological characteristics of different phenotypes based on the presence of comorbid ADHD and/or ASD and gender in a sample of youth with tic disorders through the administration of semi-structured scales and questionnaires; and (2) to evaluate symptoms variation over time and potential clinical predictors of positive treatment response in a subgroup of patients treated with Aripiprazole.

## 2. Materials and Methods

### 2.1. Study Design

This study was a naturalistic observational longitudinal study, which lasted about one year, starting from 15 December 2022. During this period, patients referred to the Department of Developmental Psychiatry and Psychopharmacology at the IRCCS Stella Maris Foundation hospital were recruited. Patients were selected based on a diagnosis of TS or chronic motor or vocal tics disorder. Questionnaires and semi-structured interviews were administered to the patients and their parents to assess their symptomatology and investigate the presence of psychiatric comorbidities. Inclusion criteria were as follows: (a) diagnosis of chronic tic disorder or TS according to DSM-5-TR diagnostic criteria; (b) Full-Scale IQ or General Ability Index > 70; (c) age between 6 and 18 years; (d) absence of syndromic/genetic pathologies. A subsample of patients was then reassessed through the administration of same clinical measures used at baseline to evaluate symptom variation over time and to identify potential predictors of response. The study was conducted in accordance with the Declaration of Helsinki and approved by the Regional Ethical Committee of Meyer Hospital (Florence, Italy) (protocol code 130/2023, approved on 23 May 2023). Informed consent was obtained from all subjects and their parents or guardians involved in the study.

### 2.2. Diagnostic Procedure and Clinical Measures

As part of our clinical routine, psychiatric conditions were diagnosed according to the Diagnostic and Statistical Manual of Mental Disorders, Fifth Edition-TR (DSM-5-TR) [1]. Diagnostic procedures were based on medical history, clinical observations, and a semi-structured interview, the Kiddie Schedule for Affective Disorders and Schizophrenia for School-Age Children-Present and Lifetime Version (K-SADS-PL) [44], administered by trained child neuropsychiatrists to the parents of all patients. Diagnoses were ultimately confirmed by the consensus of a multidisciplinary team.

All patients also underwent cognitive assessment with the Italian version of the Wechsler Intelligence Scale for Children—fourth edition (WISC-IV) [45]. In cases where ASD was suspected according to the DSM-5-TR criteria, specific psychological assessments were conducted, including the Autism Diagnostic Observation Scale—second version (ADOS-2) [46] administered to the patients and the Autism Diagnostic Interview—revised version (ADI-R) [47,48] administered to the parents.

A battery of clinician, parent, and patient-rated scales and questionnaires was administered, including the following:

- Clinic Global Impression-Severity (CGI-S) [49]: a clinician-completed global scale that assesses the overall severity of the disorder, regardless of its psychopathological complexity.
- Children’s Global Assessment Scale (CGAS) [50]: a scale completed by the clinician that evaluates the level of functional impairment.
- Yale Global Tic Severity Scale (YGTSS) [51]: semi-structured interview administered by the clinician to the patient that evaluates motor and vocal tics in the previous week (the severity of tics by analyzing number, frequency, intensity, complexity, and interference and the degree of stress and disability experienced). Five scores are obtained: total motor tic score, total vocal tic score, total tic score, global impairment score, and global severity score; internal consistency of the scale was good, both for the motor tic subscale (0.84) and the phonic tic subscale (0.90), while it was moderate for the whole scale (0.58), and the test-retest reliability was good (0.84) [52].
- Premonitory Urge for Tics Scale (PUTS) [53] investigates the presence and frequency of “premonitory sensations” by evaluating their intensity. It is a self-assessment scale consisting of nine items (>8 years); the scale demonstrated good internal consistency (0.84) and two-week test-retest reliability (0.76), as well as significant correlations with the YGTSS subscales [54].
- Child Yale–Brown Obsessive Compulsive Scale (CYBOCS) [55]: a semi-structured interview administered by the clinician to the patient that assesses the severity of obsessions and compulsions; the scale showed good internal consistency and test-retest reliability (0.81 and 0.82, respectively), and an acceptable level in the two-factor structure (obsession and compulsive) [56].
- Child Behavior Checklist 6–18 (CBCL 6–18) [57], completed by parents. Includes eight different syndrome scales, a Total Problems score, and two broad scores defined as Externalizing and Internalizing Problems; the scale has been translated and adapted in several countries all over the world, and its psychometric properties are available for many adaptations with moderate to good levels of internal consistencies of its subscales and content validity.
- Conners’ Parent Rating Scale-Revised—short form (CPRS-R) [58]: a standardized questionnaire administered to parents for the assessment of ADHD and related issues in children/adolescents (3–17 years); previous studies showed satisfactory factor structure, test-retest reliability, internal consistency, and convergent validity for all the subscales [59].
- Reactivity Intensity Polarity Stability Questionnaire—youth version (RIPoSt-Y) [60]: a self-assessment questionnaire (>11 years) that explores the emotional dimension, specifically emotional dysregulation; test-retest was significant for each subscale with moderate-to-good correlations, and internal consistency showed good-to-excellent coefficients; construct, concurrent and convergent validity was supported by significant associations other measures [60,61].
- Social Communication Questionnaire—lifetime version (SCQ) [46]: parent-completed questionnaire that investigates the subject’s socio-communication abilities during the first 4–5 years of life; the scale had excellent internal reliability and satisfactory psychometric properties that can be used for differentiating children with ASD from typical children [62].

After the introduction of pharmacological therapy with Aripiprazole in a subgroup of patients with more severe tic symptoms, the following measures were re-administered: CGI-S, CGI-I, C-GAS, YGTSS, PUTS, CBCL 6–18.

Thus, these instruments were used to assist clinicians in the diagnostic procedure and to confirm diagnoses with the final consensus of a multidisciplinary team, as regularly performed in our department according to international guidelines. Evaluations were conducted by highly experienced psychologists trained to conduct neuropsychological assessments of children.

### 2.3. Statistics

Means and standard deviations (SDs) of clinical variables with continuous distribution were computed, as well as raw numbers and percentages of nominal variables. A series of general linear models (GLMs) were run for each of the explored clinical variables at baseline as outcomes, using gender, the presence of comorbid ADHD and ASD, and their interaction as covariates. Significant associations (*p* < 0.05) were then corrected for multiple comparisons by using the Bonferroni method (*p* < 0.0008). To explore the trend of clinical variables over time during the follow-up period, clinical scores before and after follow-up were compared using a Wilcoxon test for repeated measures. Finally, a series of logistic regressions were carried out to identify potential clinical predictors of response to treatment during the follow-up period; a nominal variable obtained by labeling patients as “responders” or “non-responders” according to the score difference in the YGTSS severity scale, respectively, lower or higher than the median difference, was used as response variable. Statistical analyses were performed with RStudio^®^ software (version R 4.0.2).

## 3. Results

### 3.1. Clinical Features of Patients

Our sample of 95 patients included 78 with a diagnosis of TS and the remaining with chronic motor tic disorder. In Table 1, the main clinical features of our patients are summarized. Table 2 summarizes their clinical interventions at recruitment (T0).

Table 3 shows the average scores obtained by the sample at the clinical measure administered at baseline. Regarding questionnaires investigating tic symptoms, on the YGTSS, 37 subjects (39%) received high scores, while on the PUTS, 24 patients (28%) received low–mild scores, while 61 (72%) had moderate–high scores. The CBCL showed that the scales with the greatest prevalence of clinical scores were the following: thought problems (46 patients; 48.42%), total problems (37 patients; 38.95%), internalizing problems (34 patients; 35.79%), anxious/depressed (31 patients; 32.63%), aggressive behaviors (25 patients; 26.32%), withdrawn/depressed (24 patients; 25.26%), and social problems (24 patients; 25.26%). On the CYBOCS questionnaire, 28 patients (29.46%) received mild–moderate–severe disorder scores. On the SCQ questionnaire, 31 patients (32.63%) scored intermediate (8–15), and 12 (12.63%) scored high (>15), falling in the clinical range for ASD. In cases of suspected ASD, the ADOS-2 scale was also administered (35 patients); the majority of patients (19 patients, 54.28%) obtained a comparison score of 4–5 (moderate level of symptoms). To evaluate symptoms associated with ADHD, the CPRSR-S questionnaire was administered: 40 patients (42.11%) scored in the clinical range on the ADHD Index scale. To assess the emotional dysregulation profile, the RIPoSt-Y questionnaire was administered to 42 patients (>11 years). The majority of patients (23 patients, 54.76%) scored in the clinical range on the emotional reactivity scale.

### 3.2. Impact of ADHD/ASD Comorbidity and Gender on Tics

To evaluate the impact of the comorbidity with ADHD and/or ASD as well as of the gender on the clinical features of our patients, a series of GLMs was carried out as described above. The presence of comorbid ADHD was significantly associated with a diagnosis of TS (β = 2.75; *p* = 0.0151), as well as with the presence of comorbid OCD (β = 1.63; *p* = 0.0181), ODD (β = 2.49; *p* = 0.0406), and externalizing disorders (β = 6.19; *p* = 0.0151). Comorbid ADHD was also associated with lower PSI (β = −18.12; *p* = 0.0062) and FSIQ (β = −16.69; *p* = 0.0246) scores on the WISC-IV scale, as well as with a lower level of functioning on the C-GAS (β = −6.71; *p* = 0.0001). A significant association was also found with higher scores on the social problems (β = 6.58; *p* = 0.0048), thought problems (β = 4.98; *p* = 0.0417), attention problems (β = 11.39; *p* < 0.0001), rule-breaking behaviors (β = 6.60; *p* = 0.0007), aggressive behaviors (β = 10.59; *p* < 0.0001) scales, the internalizing problems (β = 5.63; *p* = 0.0408), externalizing problems (β = 12.35; *p* < 0.0001) and total problems (β = 11.34; *p* < 0.0001) scales, and the Dysregulation Profile Index (DPI) (β = 27.09; *p* < 0.0001). Positive associations were also observed with higher scores on the subscales of the CYBOCS, including obsessions (β = 2.79; *p* = 0.413), compulsions (β = 2.56; *p* = 0.0445), and total score (β = 5.32; *p* = 0.0338). Finally, comorbid ADHD was significantly associated with higher scores on the four subscales of the CPRS-R (β = 17.73 in “Oppositionality”; β = 19.70 in ”Inattention”, β = 17.83 in “Hyperactivity”, β = 24.06 in “Total Score”; *p* < 0.0001), while no significant associations were reported for all the other variables. After correction for multiple comparisons (*p* < 0.0008), only the following associations survived, including externalizing comorbidities, C-GAS scores, attention problems, rule-breaking behaviors, aggressive behaviors, externalizing and total problems scales and DPI of the CBCL, as well as all the subscales of the CPRS-R.

The presence of comorbid ASD was significantly associated with a lower age (β = 28.28; *p* = 0.0471) as well as a lower level of functioning on the C-GAS (β = −6.94; *p* = 0.0047). As for the CBCL, a positive association was observed with higher scores on the DPI (β = 22.96; *p* = 0.0116) and the internalizing problems (β = 11.60; *p* = 0.0039), externalizing problems (β = 6.87; *p* = 0.0483) and total problems (β = 9.96; *p* = 0.0021) scales. Comorbid ASD was significantly associated with higher scores on the ADOS-2 scale (β = 1.68; *p* < 0.0001), while no significant associations were reported for all the other variables. After correction for multiple comparisons (*p* < 0.0008), the only association that survived was with ADOS-2 scores.

The interaction between comorbid ADHD and ASD was significantly associated with a lower age (β = −27.27; *p* = 0.0288) and the diagnosis of TS (β = −2.03; *p* = 0.0301), as well as a lower level of functioning on the C-GAS (β = 7.02; *p* = 0.0159). As for the CBCL, a positive association was observed with higher scores on the anxious/depressed (β = −12.32; *p* = 0.0157) and withdrawn/depressed (β = −9.76; *p* = 0.0242) scales, the internalizing problems (β = −10.86; *p* = 0.0225) and total problems (β = −9.05; *p* = 0.0178) scales. Finally, a significant association was also reported with higher scores on the ADOS-2 scale (β = 0.71; *p* = 0.0278), while no significant associations were reported for all the other variables. After correction for multiple comparisons (*p* < 0.0008), none of the explored associations with the comorbid ADHD/ASD interaction survived.

Male gender was significantly associated only with higher scores on the withdrawn/depressed subscale (β = 5.89; *p* = 0.0347), which did not survive after correction for multiple comparisons. No significant associations were reported for all the other variables.

### 3.3. Response to Pharmacological Treatment and Clinical Predictors of Response

**Figure 1:**
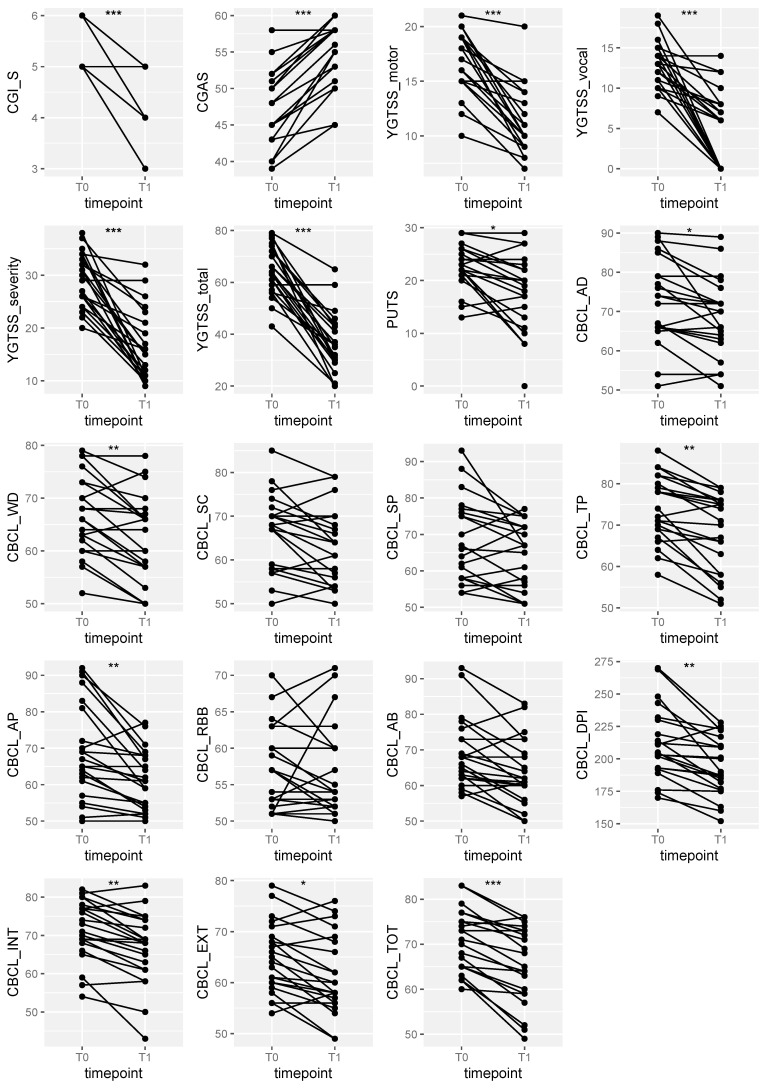
Clinical response to Aripiprazole. * *p* < 0.05; ** *p* < 0.005; *** *p* < 0.001.

A subsample of our patients prescribed with Aripiprazole was finally reassessed in follow-up. This subsample included 22 patients (23.16%), and the average duration of follow-up was 6.54 ± 2.84 months (range 2–11). The average CGI-I score in the subgroup was 2.41 ± 0.59. Clinical scores before and after follow-up were compared using a Wilcoxon test for repeated measures. Both C-GAS and CGI-S scores significantly improved during the follow-up. Particularly, a significant increase in C-GAS scores (7.14 ± 3.37; *p* < 0.0001) as well as in CGI-S scores (1.54 ± 0.59; *p* < 0.0001) was reported. The average CGI-I scores were also notably low (2.41 ± 0.59). Analyses showed significant reductions in the total YGTSS score and all its subscales, such as the motor tic scale (5.64 ± 3.12; *p* < 0.0001), the vocal tic scale (7.95 ± 5.42; *p* < 0.0001), the severity scale (11.82 ± 6.78; *p* < 0.0001), and the total score scale (30.14 ± 13.19; *p* < 0.0001), and also in the PUTS scale (*p* = 0.0017). Most subscales of the CBCL showed significant reductions, including the anxious/depressed (4.14 ± 6.13; *p* = 0.0019), withdrawn/depressed (4.50 ± 4.65; *p* = 0.0009), thought problems (5.45 ± 4.38; *p* = 0.0001), attention problems (7.27 ± 8.30; *p* = 0.0004) scales, the internalizing problems (4.73 ± 4.32; *p* = 0.0002), externalizing problems (3.55 ± 4.17; *p* = 0.0014), and total problems (5.36 ± 4.12; *p* < 0.0001) scales, and the DPI (17.54 ± 17.16; *p* = 0.0001). Trends in clinical scores over time are shown in Figure 1.

Predictors of response to pharmacological treatment were assessed using a series of logistic regressions as described above. Comorbid ADHD was the only significant negative predictor of response that emerged from the analyses (β = −2.49; *p* = 0.0402).

## 4. Discussion

This study contributes to the growing body of research on TS and the spectrum of its comorbidities, including those with other neurodevelopmental disorders such as ADHD and ASD. In our sample of patients, we described the clinical features associated with specific neurodevelopmental comorbidities and evaluated the overall changes in the clinical profile in response to the pharmacological treatment with Aripiprazole.

As widely documented [9,10], we found that TS often occurs with comorbid disorders, especially ADHD and ASD. Compared with data from the literature, in our sample, anxiety disorders and OCD appeared more represented, and more than half of the patients presented moderate–severe premonitory symptoms.

As widely reported in the literature and demonstrated in our study, patients with tic disorders comorbid with ASD and/or ADHD exhibit poorer emotional-behavioral functioning than patients with tic disorders without such comorbidities [11,12]. As reported in other studies, we found that the presence of ADHD correlated with a higher rate of OCD [13,63]. Additionally, in patients with TS and ADHD, a positive association between ADHD and externalizing disorders was observed, as reported by the significantly higher scores in the scales of CBCL related to externalizing symptoms (rule violations/aggressive behavior/emotional-behavioral dysregulation). A predominance of externalizing symptoms in ADHD patients was widely documented in the literature [64]. Conversely, patients with TS and ASD presented higher scores in the scales of the CBCL related to internalizing symptoms (anxiety/depression/withdrawal). A predominance of internalizing symptoms in ASD patients was documented in the literature [65]. The interaction of ADHD–ASD was found to be correlated with higher scores in the internalizing scales of the CBCL. Likely, the presence of TS and ADHD makes ASD have a greater impact in defining the clinical profile. At the same time, the ADHD–ASD interaction was positively associated with higher scores on the ADOS-2 scale.

Tic disorders are more frequent in the male population than in females [2,3,4,5]. Only a few studies evaluated gender differences in TS, and mainly in adult populations. Girls with TS seem to have a later peak of symptoms, less remission with age, and worsening tic symptoms, especially in adulthood, with greater interference in global functioning. About comorbidity, women show a higher rate of depression and anxiety [33], while men present more frequently externalizing symptoms, which correlate with a higher prevalence of ADHD [31,32]. In our study, we found a positive association between the male gender and high scores in “withdrawal/depression” on the CBCL. We would speculate that female youth with TS are less compromised on the psychopathological level compared to males, presenting a later peak of symptoms and an aggravation of the overall picture in adulthood [30,33]. In childhood, males would also tend to have a higher prevalence of ASD and ADHD [66,67,68].

As reported in the literature, our results provide further evidence of the role of Aripiprazole in the treatment of co-existing conditions with TS [34,37,42,43], as well as of the tic disorder itself. Nonetheless, only a few studies evaluated the potential predictors of response to treatment in patients with TS or chronic tic disorders and mainly to behavioral treatment [69,70]. In our study, we found that, in patients with TS, the presence of ADHD would negatively predict response to treatment with atypical antipsychotics. Several pharmacological trials have assessed medication for co-existing ADHD in TS. Psychostimulants, including methylphenidate (MPH) and amphetamines, are the gold standard for the pharmacological treatment of ADHD [71,72]. Some studies documented a reduction of tics in children with tics plus ADHD treated with methylphenidate [35,36]. At the same time, according to an open-label study, Aripiprazole results in an effective reduction of tics in Tourette patients with comorbid ADHD but affects ADHD symptoms only moderately [38], while Gerasch et al. suggested that Aripiprazole may play a role in improving ADHD, OCD, or affective symptoms in these subjects [37].

Our study shows that patients with tic disorders plus ADHD would present more severe symptoms and lower responsiveness to specific pharmacological treatment for tic disorders, identifying ADHD as a possible negative predictor of aripiprazole response in reducing tics.

Our preliminary findings must be viewed in light of several limitations. First, the total sample size and the subset of patients treated with Aripiprazole are relatively small for a study, and the sample size was not predetermined based on power analysis, which may limit the generalizability and statistical power of the findings. Second, about the subsample treated with Aripiprazole, we did not have a healthy control group or a control group receiving a placebo in a double-blind design. Third, the information obtained from the questionnaires presents the limits of all the self-report measures. Fourth, a comparison of clinical outcomes at specified periods after the commencement of treatment would be a better indicator of effectiveness than an average follow-up period, making it easier for the study to be compared with other published studies and replication by future researchers. All these limitations in sample size, follow-up consistency, and scope of predictors may have influenced the believability and generalizability of our findings.

## 5. Conclusions

Our study confirms that tic disorders, associated with other neurodevelopmental disorders, are characterized by worse emotional and behavioral functioning. Based on the most frequently occurring disorders in comorbidity with tic disorders, ADHD and ASD, the study attempts to outline different clinical phenotypes. It was highlighted that tic disorders comorbidity with ADHD could be associated with a greater expression of externalizing symptoms, while tic disorders comorbidity with ASD could be characterized by more internalizing symptoms. The interaction between ADHD and ASD could be associated with internalizing symptoms and more expression of autistic symptoms.

The study also attempted to assess the influence of gender on the overall clinical expression. The absence of clinical differences, except for higher scores in males on the “withdrawal/depression” scale of the CBCL, could suggest that the female gender may act as a protective factor. Finally, pharmacological treatment with Aripiprazole was observed to result in overall clinical improvement of outcome beyond just tic symptoms, although our naturalistic observational study with the limitations discussed above was not primarily intended to evaluate the effectiveness of this pharmacological intervention. Nonetheless, among the predictors of response, the presence of comorbid ADHD appears to be associated with a worse response of Aripiprazole to tic symptoms.

## Figures and Tables

**Table 1:** Clinical features of the sample.

	Mean ^a^ or Number ^b^	SD ^a^ or Percentage ^b^	Range
*Gender (M)*	80 **^b^**	84.2% **^b^**	-
*Age (years)*	11.10 **^a^**	2.11 **^a^**	6.00–17.9
*Tourette syndrome*	78 **^b^**	82.10% **^b^**	-
*Age of onset of motor tics (years)*	6.04 **^a^**	1.66 **^a^**	2–10.4
*Age of onset of complex motor tics (years)*	6.9 **^a^**	24.93 **^a^**	4–13
*Age of onset of vocal tics (years)*	7.25 **^a^**	26.45 **^a^**	3–16
***Comorbidities***			
*ADHD*	56 **^b^**	58.95% **^b^**	-
*ASD*	34 **^b^**	35.79% **^b^**	-
*OCD*	33 **^b^**	34.74% **^b^**	-
*GAD*	61 **^b^**	64.21% **^b^**	-
*BSD*	25 **^b^**	26.32% **^b^**	-
*Dep*	7 **^b^**	7.37% **^b^**	-
*IED/ICD*	14 **^b^**	14.73% **^b^**	-
*ODD*	14 **^b^**	14.73% **^b^**	-
*CD*	1 **^b^**	1.05% **^b^**	-
*SLD*	19 **^b^**	20% **^b^**	-
***WISC-IV***			
*VCI*	105. 36 **^a^**	18.97 **^a^**	68–156
*PRI*	106.60 **^a^**	19.74 **^a^**	65–148
*WMI*	97.42 **^a^**	20.93 **^a^**	58–148
*PSI*	87.84 **^a^**	19.58 **^a^**	50–144
*GAI*	98.00 **^a^**	17.39 **^a^**	66–134
*CPI*	79. 96 **^a^**	16.60 **^a^**	48–113
*FSIQ*	99.30 **^a^**	20.80 **^a^**	53–154

Legend: GAD: generalized anxiety disorder; BSD: bipolar spectrum disorders, Dep: depression, IED/ICD: intermittent explosive disorder/impulse control disorders, ODD: oppositional defiant disorder, CD: conduct disorder; SLD: specific learning disorders, VCI: Verbal Comprehension Index, PRI: Perceptual Reasoning Index; WMI: Working Memory Index, PSI: Processing Speed Index, GAI: General Ability Index, CPI: Cognitive Proficiency Index, FSIQ: Full Scale IQ. ^a^ Means and SDs are provided for variables with continuous distribution; ^b^ raw numbers and percentages are provided for variables with nominal distribution.

**Table 2:** Clinical interventions of the sample.

	Number	Percentage
***Medications***	16	16.84%
*Lithium*	1	1.05%
*AED*	3	3.15%
*SGA*	7	7.37%
*SSRI*	5	5.26%
*Stimulants*	5	5.26%
*FGA*	1	1.05%
***Psychotherapy***	23	24.2%

Legend: AED: antiepileptic drugs; SGA: second-generation antipsychotics, SSRI: selective serotonin reuptake inhibitors, FGA: first-generation antipsychotics.

**Table 3:** Scales administered to the sample with tic disorder.

	Mean	SD
***CGI-S***	4.82	4.70
***C-GAS***	50.77	33.70
***YGTSS***		
*Motor Tics*	13.71	4.46
*Vocal Tics*	9.15	6.10
*Severity*	22.73	9.60
*Total Score*	46.01	20.96
***PUTS***	18.13	10.85
***CBCL***		
*Anxious/depressed*	65.53	9.66
*Withdrawn/depressed*	62.44	9.67
*Somatic complains*	61.42	8.97
*Social problems*	63.40	8.97
*Thought problems*	68.15	8.99
*Attention problems*	64.02	10.17
*Rule breaking behavior*	57.79	7.13
*Aggressive behavior*	63.93	9.69
*Emotional dysregulation profile index*	193.40	25.36
*Internalizing problems*	64.60	10.29
*Externalizing problems*	61.32	9.79
*Total problems*	65.37	9.20
***CYBOCS***		
*Obsessions*	3.34	3.32
*Compulsions*	3.32	4.48
*Total*	6.65	8.92
***SCQ-Lifetime***	7.83	5.38
***ADOS-2***	5.17	1.33
***CPRS-R***		
*Oppositionality*	63.21	15.97
*Cognitive problems/inattention*	63.38	17.38
*Hyperactivity*	62.15	17.43
*ADHD Index*	65.09	16.85

Abbreviations: ADOS, Autism Diagnostic Observation Scale; C-GAS, Children Global Assessment Scale; CBCL, Child Behavior Checklist; CGI-S, Children Global Impression Scale; CPRS, Conners’ Parent Rating Scale; CYBOCS, Children Yale–Brown Obsessive Compulsive Scale; PUTS, Premonitory Urge for Tics Scale; SCQ, Social Communication Questionnaire; YGTSS, Yale Global Tic Severity Scale.

## Data Availability

The raw data supporting the conclusions of this article will be made available by the authors without undue reservation.
